# Perceptions of ophthalmologists on the impact of trachoma in Egypt: a mixed-methods, nationwide survey

**DOI:** 10.1186/s12879-022-07862-w

**Published:** 2023-01-17

**Authors:** Yassin Nayel, Matilda Taylor, Ahmed S. Montasser, Mohamed Elsherif, Mostafa M. Diab

**Affiliations:** 1 American University of the Caribbean School of Medicine, Cupecoy, Sint Maarten; 2 Faculty of Medicine, University of British Columbia, Vancouver, BC Canada; 3 Department of Ophthalmology, Faculty of Medicine, Helwan University, Helwan, Egypt; 4 Department of Epidemiology and Population Health, Faculty of Health Sciences, American University of Beirut, Beirut, Lebanon; 5 Department of Ophthalmology, Faculty of Medicine, Fayoum University, Al Fayoum, Egypt

**Keywords:** Trachoma, Trichiasis, Egypt, Survey, Ophthalmologists’ perception

## Abstract

**Purpose:**

Understanding the perception and practices of ophthalmologists for trachoma is important to develop interventions aimed at disease elimination in Egypt. The survey investigated: (1) the views and practice patterns of Egyptian ophthalmologists for trachoma and (2) the influence of geographic location, setting, and years of practice on ophthalmologists’ perceptions.

**Methods:**

A questionnaire sent to ophthalmologists currently working in Egypt collected information on: (1) demographics, (2) caseload and practice patterns for trachoma, (3) 13 Likert scale questions regarding the current state of trachoma, and (4) two open-ended written response questions.

**Results:**

Of the 500 recipients, 194 ophthalmologists participated. 98% of the respondents reported seeing trachoma patients in their practice. 28.8% agreed that trachoma is currently an active health problem in Egypt, with ophthalmologists in public practice having significantly higher agreement scores compared to private practitioners (p = 0.030). Rural ophthalmologists were significantly more likely to agree that a targeted trachoma control program is needed in their location of practice compared to their urban counterparts (p < 0.001). Open-ended questions revealed recurrent themes, including the rural distribution of trachoma patients and the high volume of patients with corneal opacity.

**Conclusion:**

Ophthalmologists’ experiences with trachoma in Egypt differed based on practice setting, years in practice, and location, and the overall perception of the impact of the disease remains low. However, there was widespread agreement that trachoma is present in communities across the country. Practitioners in rural areas and in the public sector shared a disproportionate burden of the trachoma caseload. The perspectives of such ophthalmologists must be emphasized in decision-making related to trachoma interventions.

## Introduction

Trachoma is the leading infectious cause of blindness [1]. Interventions, such as the Global Trachoma Mapping Project (GTMP) and the implementation of the Surgery, Antibiotics, Facial Cleanliness, Environmental Improvement (SAFE) strategy have played an important role towards the ongoing goal of the global elimination of trachoma [2–4].

According to the World Health Organization’s (WHO) latest progress report, trachoma remains a current health problem in Egypt that is known to require intervention [5]. Since the commencement of the GTMP in 2012, four districts in Egypt have been mapped out of a total of 29 districts where trachoma is expected to be endemic [6]. There is a relative lack of effort toward the elimination of trachoma in Egypt with no existing national trachoma-targeted control programs [7]. Instead, most patients are treated on an individual basis rather than as part of a larger public health program [7]. The only mass drug administration (MDA) following the mapping of the four districts came in 2019 and was carried out by the Egyptian Ministry of Health, district-level governments and international organizations, which distributed azithromycin to 300,000 residents [8]. There are no available follow-up data on the efficacy of this project and no further MDAs have been conducted since.

When considering trachoma elimination in Egypt, it is critical to understand the local context [9]. Egypt has a relatively high number of practicing ophthalmologists, estimated at 2400 (2.2 per 100,000 people) [10]. Egypt is also unique in that trichiasis surgeries must be performed by licensed ophthalmologists [11]. This differs from the trachoma elimination programmes implemented in most other countries, where trained eye nurses perform surgeries for trichiasis [12]. Due to these factors, in Egypt, ophthalmologists are the primary care providers for patients with trachoma.

Ophthalmologists frequently encounter trachoma patients, allowing them to provide targeted insight into the disease. Due to their unique position, their understanding of disease prevalence, barriers to elimination, and treatment methods is important to ascertain in the effort to eliminate the disease and to develop comprehensive control programs. This is especially true in a setting with limited baseline mapping [13]. In this study, we surveyed Egyptian ophthalmologists aiming to investigate their views and practice patterns with regards to trachoma, and to evaluate the influence of the geographic location, years of practice, and practice setting on their perceptions.

## Methods

We conducted a cross-sectional, self-administered questionnaire-based study. The questionnaire was pretested in a pilot study with 35 participants. The questions were revised based on feedback. A link to an anonymous and confidential survey hosted on Google Forms was distributed by email and social media to 500 ophthalmologists currently working in the private and public sector in Egypt including residents, specialists, and consultants. These 500 ophthalmologists were contacted because they are the only practitioners with available contact information at the Egyptian Ophthalmological Society. The questionnaire was made available for responses from June 15, 2021 to June 25, 2021. Ethical approval was obtained from Helwan University Scientific Research Ethics Committee (Reference number: 29-2021). The study adhered to the principles of the Declaration of Helsinki.

The questionnaire included five sections. The first section collected demographic data including age, gender, sub-specialty training, workplace (governorate), working sector (private/university/governmental hospital) and years of ophthalmology practice. The second section focused on caseload and practice patterns for trachoma. The third section assessed perceptions using 9 Likert scale questions related to the current state of trachoma in Egypt. The fourth section consisted of 4 Likert scale questions related to the SAFE strategy and its suitability for Egypt. The fifth section included two open-ended, optional written response questions, asking respondents to explain why they consider or do not consider trachoma an issue in Egypt.

### Data processing and analysis

Descriptive statistics were presented in the form of mean (SD) for normally distributed numeric variables, median (IQR) for non-normally distributed numeric variables, and frequencies and percentages for categorical variables. The comparison of perception and SAFE strategy scores across different groups of ophthalmologists was done using independent t-test and one-way ANOVA. The comparison of Likert-scale questions was done using the Mann–Whitney and Kruskal Wallis tests. IBM SPSS statistics software, version 26, was used for the analysis and a p-value < 0.05 was considered statistically significant. Perception scores were calculated for 13 questions. Each question was graded on a five-point Likert scale, which ranged from “1” for strongly disagreeing to “5” for strongly agreeing. “3” was neutral (Table 2). These scores were used to compare participants according to demographics, location or setting of practice. The final written responses were thematically analyzed using NVivo 1.3 software for data coding. The coding scheme was derived from scanning the data for emergent ideas and patterns. A code book was generated to organize and group main nodes and sub nodes into clusters and definitions were determined [14]. A preliminary codebook was reviewed, and the changes applied. The coded data were analyzed to establish themes and the significance in correlation with other survey data.

## Results

**Fig. 1:**
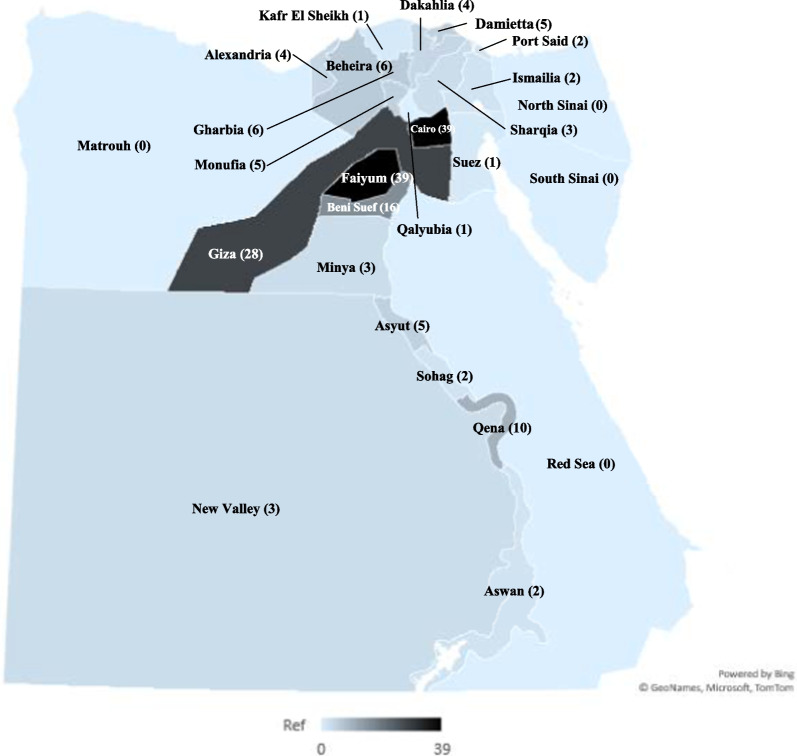
Number of questionnaire respondents by governorate

We received 194 responses with a response rate of 38.8%. Seven responses were excluded from the analysis for not meeting criteria. Survey responses arrived from 22 out of a total of 27 governorates (Fig. 1). 89 (47.6%) of the respondents see most of their patients in the private sector, while 68 (36.4%) in public and 30 (16.0%) in university hospitals. 113 (60.4%) respondents practice ophthalmology in rural areas. The descriptive data of the participants is presented in Table 1.

**Table 1:** General demographics of participants

Respondents’ characteristics	n (%)
Gender	
Male	121 (64.7)
Female	66 (35.3)
Age	
20–40	108 (57.8)
40–60	70 (37.4)
60 and over	9 (4.8)
Location of practice	
Urban	74 (39.6)
Rural	113 (60.4)
Setting of practice	
Private practice	89 (47.6)
Public/Governmental Eye Hospital	68 (36.4)
University Hospital	30 (16)
Subspecialty training	
Cornea	31 (16.6)
Glaucoma	9 (4.8)
Oculoplastic	28 (15)
Retina	24 (12.8)
Other	29 (15.5)
No subspecialty training	66 (35.3)
Years of practice	
0–5 years	30 (16)
5–15 years	84 (44.9)
15 and over	73(39)

### Trachoma caseload

**Fig. 2:**
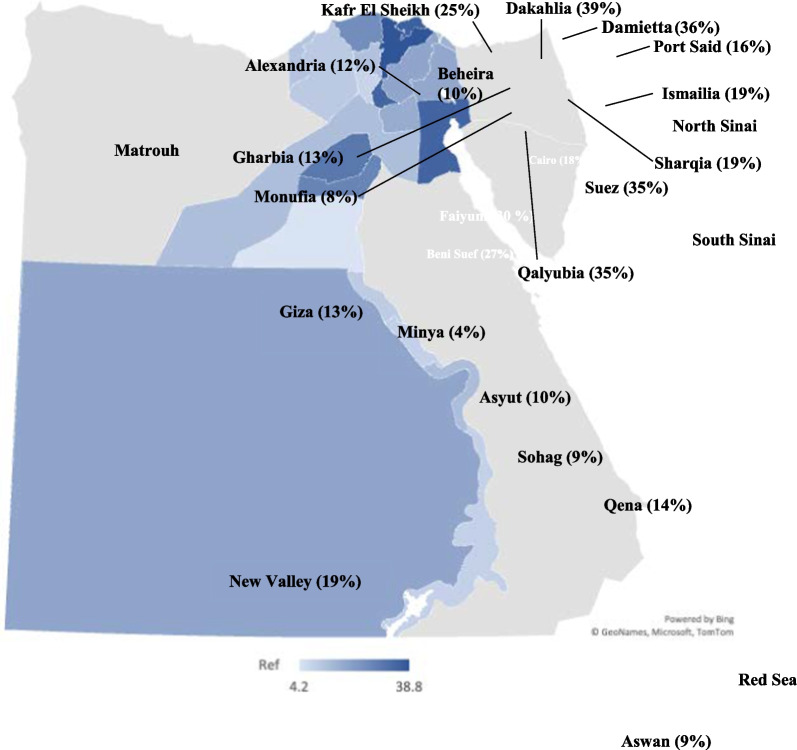
Mean percentage of responses to “Of your total caseload, what percent have trachoma?” by governorate. (Grey indicates no data from the governorate)

184 (98.4%) of the respondents reported seeing trachoma patients in their practice (Fig. 2). Out of five available choices (cataract, glaucoma, trachoma, diabetic retinopathy, and errors of refraction), trachoma was the third most common disease seen by the respondents after errors of refraction and cataracts. Trachoma represented 10–50% of the total caseload of 50% of the respondents. According to 43.8% of the respondents, 10–50% of their trachoma patients required trichiasis surgery.

### Practice patterns

All the participants reported that they rely on clinical examination to diagnose trachoma without confirmatory tests. 2 (1.1%), 11 (5.9%), 13 (7.0%), 25 (13.4%), and 134 (71.7%) of the respondents used topical tetracycline ointment, topical tobramycin/dexamethasone, azithromycin eye drops, oral azithromycin, and a mixture respectively for treating active disease.

For minor trichiasis^1^ (≤ 5 lashes), 143 (76.5%) recommended epilation and 128 (68.4%) performed epilation themselves. For patients requiring trichiasis surgery, 99 (52.9%) of the respondents refer cases to a specialist.

### Perception of current state of trachoma

Participants graded their responses regarding the current state of trachoma in Egypt on a 5-point Likert scale. 28.8% agree or strongly agree that trachoma is a current health problem in Egypt compared to 36.3%, who disagree or strongly disagree. 24.6% consider blinding trachoma endemic in Egypt, while 42.3% do not. 35.3% consider trachoma a neglected disease in Egypt. Despite the perception that trachoma is not a problem, 78.6% believed that a trachoma control program is needed in their location of practice and 68.4% feel that more resources should be allocated towards the elimination of trachoma in Egypt (Table 2).

**Table 2:** Responses to Likert scale questions

		Strongly disagree	Disagree	Neutral	Agree	Strongly agree	Average agreement score
In the past, trachoma was an active health problem in Egypt	N	9	3	17	55	103	4.28
	%	4.8	1.6	9.1	29.4	55.1	
Trachoma is currently an active health problem in Egypt	N	10	58	65	50	4	2.89
	%	5.3	31.0	34.8	26.7	2.1	
Blinding trachoma is endemic in Egypt	N	16	63	62	35	11	2.80
	%	8.6	33.7	33.2	18.7	5.9	
Trachoma patients mainly live in rural, underserved areas	N	11	7	21	93	55	3.93
	%	5.9	3.7	11.2	49.7	29.4	
I believe that more trachoma patients can be identified on screening convoys to rural areas	N	4	8	28	99	48	3.96
	%	2.1	4.3	15.0	52.9	25.7	
A targeted Trachoma Control Program is needed in your location of practice	N	11	27	60	74	15	3.29
	%	5.9	14.4	32.1	39.6	8.0	
In some cases, cost prevents patients from being able to receive treatment	N	21	45	29	70	22	3.14
	%	11.2	24.1	15.5	37.4	11.8	
Trachoma is a neglected disease in Egypt	N	14	48	59	51	15	3.03
	%	7.5	25.7	31.6	27.3	8.0	
More resources should be allocated towards the elimination of trachoma in Egypt	N	9	9	41	76	52	3.82
	%	4.8	4.8	21.9	40.6	27.8	
All patients with trichiasis in Egypt require surgical intervention regardless of severity	N	32	93	33	22	7	2.35
	%	17.1	49.7	17.6	11.8	3.7	
Community-based mass distribution of antibiotics is required for trachoma elimination in Egypt	N	19	47	53	53	15	2.99
	%	10.2	25.1	28.3	28.3	8.0	
Individualized antibiotic regimens are required for trachoma elimination in Egypt	N	5	19	52	85	26	3.58
	%	2.7	10.2	27.8	45.5	13.9	
Community-based environmental sanitary interventions are required for trachoma elimination in Egypt	N	8	9	30	83	57	3.92
	%	4.3	4.8	16.0	44.4	30.5	

### SAFE strategy

66.8% did not agree that all patients with trichiasis in Egypt require surgical intervention regardless of severity. Regarding antibiotic distribution, most participants agreed that individualized antibiotic regimens are necessary (59.4%). 74.9% believe that environmental enhancements are required for disease control.

### Subgroup analysis

**Fig. 3:**
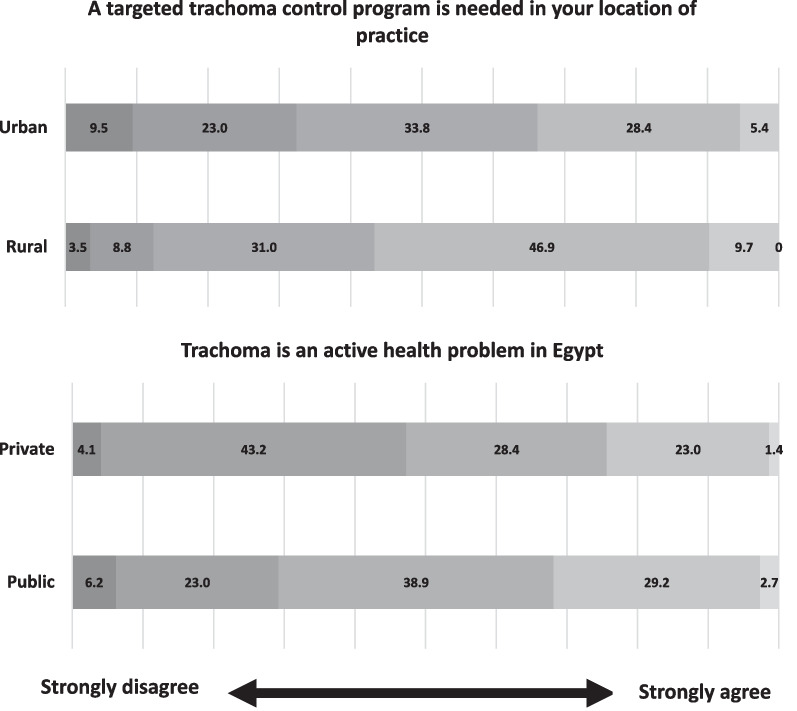
Likert scale responses of rural and urban practitioners (above) to “A targeted trachoma control program is needed in your location of practice.” Likert scale responses of private and public practitioners (below) to “trachoma is an active health problem in Egypt.” Responses range from 1 (strongly disagree) to 5 (strongly agree)

Ophthalmologists in rural areas were significantly more likely to voice a need for a targeted trachoma control program in their location of practice (median/IQR: 4/1) compared to those working in urban areas (median/IQR: 3/2) (p < 0.001) (Fig. 3). In response to “trachoma is currently an active health problem in Egypt”, ophthalmologists working in the public sector had significantly higher agreement scores (median/IQR: 4/1) than those in the private sector (p = 0.030) (Fig. 3).

### Written response results

The open-text responses further supported the results of the survey, while providing additional insight into the rationale behind the respondent’s perception of the disease. 115 participants responded to one of the questions (61.5%).

Eighty participants (42.8%) expanded on why they believe trachoma is an issue in Egypt. The most common theme was the high prevalence of the disease and its sequelae seen in practice. Many respondents noted that trachoma is one of the most common diseases they encounter, with one respondent stating:

"All patients even when they [don’t have] an active disease, they have at least one of its complications."

Prevention was frequently discussed. Many respondents mentioned the need for early detection and screening to prevent severe complications.

"The disease is diagnosed late with late treatment leading to severe irreversible complications."

Some respondents noted that the disease persists at high levels because it is neglected, underestimated, and misdiagnosed. The following respondent noted several of the key reasons for this underrepresentation:

"The number of cases is way underestimated due to, in part, to the sometimes-limited reach of medical services to rural areas as well as the inexperience of young ophthalmologists misdiagnosing active trachoma for other conditions e.g. allergic conjunctivitis, mucopurulent conjunctivitis."

A prominent theme noted was the increased burden of the disease in rural and poverty-stricken areas. Some attribute these increased levels to lower socio-economic status, which decreases patient access to health care and sanitation facilities. Some indicated a need for campaigns and convoys to treat patients in these areas.

"It is an issue in rural areas and needs localized elimination programs in some governorates."

Regarding medical interventions, multiple aspects of the SAFE strategy were mentioned. However, most comments indicated the need for increased hygiene and sanitation, especially when relating to rural and low-income areas. Several respondents discussed the need for official interventions:

"Because it is an avoidable disease, and we still see patients (especially those with poorer background[s]) I believe it is a problem that needs official intervention."

Several respondents focused on the social aspects of the disease and the need for increased education about hygiene habits and the disease in general.

Thirty-five participants (18.7%) elaborated on why they believe trachoma is not an issue. Responses highlighted simple and effective treatments, and a decreased burden of the disease compared to previous levels. One respondent noted:

"We don’t see it a lot anymore, we rarely see [the] consequences so we don’t have to spend extra money on screening or treating."

Some participants noted the need to focus on other more prevalent diseases that they encounter, as seen in the following response:

“In my practice, it wasn’t that prevalent [of an] issue, on the other hand glaucoma and diabetic retinopathy are catastrophic issues that should be preventable by targeted screening and community enlightenment.”

Many attributed decreased trachoma levels to an increased access to health care, health education, improved socioeconomic status and increased usage of neonatal antibiotics.

"[Trachoma is] not an [issue any] more, due to improved medical health[care] and early use of antibiotics."

Respondents mentioned the decreasing levels of active trachoma, however, there is still an indication that many still see trachoma sequelae in older and rural patients:

"In Cairo, blinding trachoma [cases] are not seen frequently, most patients I see are from rural areas."

## Discussion

Trachoma has long been endemic in Egypt with reports tracing the history of the disease to Ancient Egypt in 1500 BC [15, 16]. In the 1960s, the Egyptian Ministry of Health conducted a large-scale antibiotic treatment program, with the aim of eliminating trachoma as a public health problem [17]. However, in 1989 a survey from the Nile Delta found evidence of conjunctival scarring in over 90% of all 25-year olds and trichiasis in over 75% of older women [18]. The World Health Organization’s latest progress report states that trachoma is still a current health problem in Egypt that is known to require intervention [19]. Mapping has only been completed in four out of 29 districts in need of baseline mapping [6]. Three of the four districts showed a trachomatous inflammation—follicular (TF) prevalence > 10% in 1–9-year olds, with the fourth district having a slightly lower rate of TF prevalence of 5–9.9% in the same age group [6]. The burden of the disease in the country can only be estimated from these preliminary numbers.

Our findings indicate that ophthalmologists from all over the country encounter the disease regularly, with 98.4% of respondents stating that they see trachoma patients in their practice (Fig. 2). However, contrary to recent mapping and historic data, their overall perception of the impact of the disease remains low, with only 28.8% stating that trachoma is currently a public health problem. As a disease of poverty, trachoma mainly affects patients with limited access to healthcare, and thus ophthalmologists may not be seeing the full extent of the disease. This is further supported by many respondents stating that they do not see cases of active trachoma in their practice and rather mainly treat sequelae of long-standing disease. Furthermore, our findings point to a lack of understanding of trachoma as a community disease that requires community-based interventions. The WHO recommends three annual rounds of MDAs in areas where the prevalence of trachomatous inflammation—follicular is more than 10% in 1- to 9-year-olds [20]. Yet, when asked about the use of antibiotics for trachoma control, most respondents (59.4%) recommend managing trachoma patients with antibiotics individually as they present to their clinics. This approach, however, does not account for individuals who have conjunctival C. *trachomatis* infection with no clinically significant signs, and individuals with active trachoma who do not seek ophthalmic care. It also neglects antibiotic treatment in children, a key demographic targeted in MDAs to break infection cycles.

In our subgroup analysis, we noticed more urgency in addressing trachoma from practitioners in rural areas when compared to their urban counterparts. As many respondents noted, spatial inequality is ever present in Egypt. One participant who disagreed that trachoma is a current health problem pointed to this issue in the following quote: “*In Cairo, blinding trachoma [cases] are not seen frequently, even most patients I see are from rural areas*.” Poverty in rural areas is three times higher than in urban areas [21]. In upper Egypt, 50.7% of rural residents live below the poverty line [22]. Because of this disparity, along with the well-established association between trachoma and rural life [23], it is not surprising that our findings indicate that rural practitioners are significantly more likely to agree that a trachoma control program is needed in their location of practice.

Trachoma used to be common in Europe and North America, however, it disappeared with socioeconomic improvements, not as a result of trachoma control programs [24]. Many ophthalmologists who disagree that trachoma is an issue believe that something of this nature has occurred in Egypt. In our qualitative analysis, these respondents mention the decreased caseload compared to previous levels: “numbers are declining” and “it was a problem, but not anymore.” With limited historic and current mapping data [13] it is difficult to determine the validity of these claims, but it is possible that this is true in some urban areas that have seen significant economic improvements. However, the disease persists in areas of poverty, mostly affecting people in rural areas with a low socioeconomic status [25]. Our results show that most ophthalmologists are aware of these trends with 79.1% of respondents agreeing that trachoma patients in Egypt live in rural, underserved areas and 49.2% agreeing that cost can prevent patients from being able to receive treatment. In urban centers, Egyptians are better positioned to access quality private healthcare, whereas low-income Egyptians in rural areas do not have the same access and instead turn to public care, if available [26]. The findings show that ophthalmologists in public practice are significantly more likely to agree that trachoma is an active health problem, when compared to their counterparts in private practice. This is most likely attributed to ophthalmologists working in public practice having more exposure to the full spectrum of the disease.

With a rapid growth in population that has put an increased strain on the public health system [26], along with limited funding, public health care currently lacks the ability to provide for marginalized populations with trachoma on its own. The National Coordinator of Prevention of Blindness in Egypt highlighted the need for greater cooperation from NGOs and other partners [7]. Control programs must focus on targeting resources to those most affected by the disease, increasing access, and decreasing barriers to care uptake [27].

As primary providers of eye care, ophthalmologists in Egypt provide a unique perspective, and can play a major role in the effort to eliminate trachoma. A total of 187 ophthalmologists responded to the survey, approximately 7.8% of the estimated 2400 ophthalmologists currently practicing in Egypt [28]. We are encouraged by the number of respondents, especially since there is no centralized database for communicating with ophthalmologists. A centralized database would be valuable, as it would allow easier access to ask these types of questions.

Currently there is a lack of a national baseline study to estimate the extent of the disease in Egypt [13], limited mapping efforts, and no national trachoma control program [7]. Without adequate mapping, ophthalmologists must rely on their experience to determine disease severity. This becomes an issue when ophthalmologists and other health care practitioners are the ones informing health care policy. We found a statistically significant difference between subgroups based on location, setting and years of experience. As trachoma patients are concentrated in rural areas, the voices of rural ophthalmologists need to be emphasized in the development of a community-based or national trachoma targeted control program and more funding should be allocated towards public health efforts of disease elimination. Due to the overall low perception of the impact of trachoma among ophthalmologists, more information needs to be imparted to practitioners about trachoma as a public health problem that requires community-based interventions.

Our approach to survey ophthalmologists provides an important perspective of key professionals in the effort towards trachoma elimination. However, this approach also has limitations. While we received responses from 22 to 27 governorates, a disproportionate number of responses (57.8%) came from Cairo, Giza and Fayoum. This may have created a bias in our results emphasizing the experience of ophthalmologists practicing in a localized geographical region in central Egypt. Additionally, with no centralized database for communicating with ophthalmologists, we were only able to reach 500 of the estimated 2400 practitioners in the country, as these were the only ophthalmologists with contact information available at the Egyptian Ophthalmological Society. After receiving feedback through our pilot survey, we limited respondents to one answer when asked to report which governorate and setting (public or private) they spend most of their time practicing in. However, most ophthalmologists in Egypt practice in multiple locations and settings, a trend that was reflected in our pilot survey. We assumed that the responses were based on the experiences of ophthalmologists influenced by the location and setting they spend most of their time practicing in, however, responses could have been influenced by their experiences elsewhere. Also, although many participants expanded on their experience with trachoma in practice, highlighting how they mostly encounter patients with disease sequelae rather than active trachoma, a clear distinction between the two was not made in the survey questions. Finally, by solely focusing on ophthalmologists, we were unable to gain an understanding of patients’ perspective of the severity of trachoma and access to ophthalmological care, which could differ greatly. Gaining the perspective of patients and the complex issues they face when accessing care should be the focus of a future study.

## Conclusion

In summary, our survey found that most ophthalmologists in Egypt see trachoma patients, yet the overall perception of the impact of the disease remains low. More urgency in addressing trachoma as a public health issue is seen from ophthalmologists practicing in rural areas and in the public health sector. More awareness needs to be raised within the ophthalmology community with regards to trachoma being a public health problem that requires community-based interventions. This study indicates that practitioners with the highest prevalence of trachoma in their practice should be involved in policy and program development. Further involvement of ophthalmologists may result in increased prioritization of targeted elimination efforts on a national level. One of the respondents summed up these points perfectly: “trachoma is still a living issue in Egypt but is also easily treatable and definitely preventable without deploying too many resources which would spare us the trouble of dealing with more devastating complications, ‘an ounce of prevention is worth a pound of cure.’”.

## Data Availability

The datasets used and/or analysed during the current study are available from the corresponding author on reasonable request.

## Footnotes

1 Definitions: Minor trichiasis: 1–5 lashes; Rural: All governorates other than urban; Urban: Cairo, Giza, Suez, and Alexandria.
